# Rutin attenuates bleomycin-induced acute lung injury *via* miR-9-5p mediated NF-κB signaling inhibition: network pharmacology analysis and experimental evidence

**DOI:** 10.3389/fphar.2025.1522690

**Published:** 2025-03-05

**Authors:** Fatema S. Alatawi, Awatif M. E. Omran, Eman Rashad, Omnia N. Abdel-Rahman, Ahmed F. Soliman

**Affiliations:** ^1^ Biochemistry Department, Faculty of Science, University of Tabuk, Tabuk, Saudi Arabia; ^2^ Cytology and Histology Department, Faculty of Veterinary Medicine, Cairo University, Giza, Egypt; ^3^ Biological and Geological Sciences Department, Faculty of Education, Ain Shams University, Cairo, Egypt; ^4^ Biochemistry Department, Faculty of Science, Ain Shams University, Cairo, Egypt

**Keywords:** acute lung injury (ALI), bleomycin, inflammation, miR-9-5p, rutin

## Abstract

**Introduction:**

Although successfully used as a chemotherapeutic agent in various malignant diseases, acute lung injury (ALI) is one of the major limitations of bleomycin (BLM). Seeking reliable natural remedies, this study aimed to explore the potential effect of rutin on BLM-induced ALI.

**Methods:**

Targets of rutin and ALI were collected using various databases. Enrichment analyses of common targets were conducted, a protein-protein interaction (PPI) network was constructed, the hub genes were identified, and the upstream miRNA interacting with the top hub gene was later predicted. A BLM-induced ALI rat model was established to verify rutin potential effects, and the selected hub gene expression with its upstream regulatory miRNA and a downstream set of targets were examined to elucidate the action mechanism.

**Results:**

A total of 147 genes have been identified as potential therapeutic targets of rutin to treat BLM-induced ALI. Data from the enrichment and PPI analyses and the prediction of the upstream miRNAs indicated that the most worthwhile pair to study was miR-9a-5p/*Nfkb1*. *In vivo* findings showed that rutin administration significantly ameliorated pulmonary vascular permeability, inflammatory cells alveolar infiltration, induction of proinflammatory cytokines in the bronchoalveolar lavage fluid, and lung histology. Mechanistically, rutin downregulated the gene expression level of *Nfkb1*, *Ptgs2*, *Il18*, and *Ifng*, alongside their protein products, NF-κB p50, COX-2, IL-18, and IFN-γ, accompanied by an upregulation of rno-miR-9a-5p, Il10, and IL-10 expression in lung tissues.

**Conclusion:**

Combining network pharmacology and an in vivo study revealed that miR-9-5p/Nfkb1 axis could mediate the meliorative effect of rutin against BLM-induced ALI.

## 1 Introduction

Chemotherapeutic drugs have shown undesirable side effects, limiting their applications. Using natural products with chemotherapeutics can help reduce the associated toxicity and enhance therapeutic efficiency (Farha et al., 2022). Currently, bleomycin (BLM) is used, in combination with other drugs, against a broad spectrum of tumors with a high cure rate, especially in patients with testicular and metastatic ovarian cancer. However, the treatment with BLM is cursed by many side effects, the most severe of which is lung toxicity, which can affect as many as 46% of the patients under treatment (Della Latta et al., 2015; Murray et al., 2018). Toxicity starts with the induction of pneumonitis and acute lung injury (ALI) that progresses gradually to fibrosis (Shippee et al., 2016).

With a persistently high morbidity and mortality rate, ALI, and its more serious form, acute respiratory distress syndrome (ARDS), are considered severe pulmonary inflammatory diseases that describe clinical syndromes of acute respiratory failure. ALI and/or ARDS are responsible for more than 10% of all intensive care unit admissions and 4% of all hospital admissions. Moreover, data suggests that the quality of life of ALI survivors is negatively impacted in the long term (Mowery et al., 2020; Bellani et al., 2016; Dowdy et al., 2006). ALI characteristics include compromised integrity of the alveolar-capillary membrane, excessive transepithelial migration of neutrophils, and release of pro-inflammatory cytokines (5). Despite the recent progress in understanding the ALI epidemiology and pathogenesis, there is no specific therapeutic strategy for the disease yet. Therefore, more research is required to find new treatments (Johnson and Matthay, 2010).

Rutin, also known as quercetin-3-O-rutinoside and vitamin P, is a polyphenolic natural flavonoid possessing many biological activities, including antibacterial, anti-inflammatory, antioxidant, and antiapoptotic (Deepika et al., 2019; Abarikwu et al., 2022; Gęgotek et al., 2019). The preventive effect of rutin against several inflammatory diseases, such as colitis and cardiac inflammation, has been previously confirmed (Sharma et al., 2021; Oluranti et al., 2021). Further, rutin was reported to alleviate desflurane-, lipopolysaccharide (LPS)-, and ventilator-induced lung injury (Tosun et al., 2021; Huang et al., 2016; Tian et al., 2022; Chen et al., 2023). However, studies based on bleomycin as an inducing agent of lung injury are scarce and focused on the role of transforming growth factor-β signaling in lung fibrosis (Bai et al., 2020; Karunarathne et al., 2024).

Recently, integrating computational methods and experiments has proven efficient in achieving goals in natural product research, such as the objective elucidation of action mechanisms and the comprehensive prediction of potent therapeutic approaches (Kibble et al., 2015; Guney et al., 2016). In the same context, a growing body of evidence has shown that systems pharmacology could be an enlightening avenue for exploring the therapeutic mechanism of compound and herbal medicine in ALI (Li et al., 2019; Wang et al., 2022). Considering their importance as crucial regulatory molecules in ALI and gene expression networks (Jiang et al., 2020; Lu et al., 2022), studying microRNAs (miRNAs) in ALI could provide potential therapeutic targets.

Accordingly, this study aimed to investigate the potentiality of rutin against BLM-induced ALI using network pharmacology approaches.

## 2 Materials and methods

### 2.1 Collecting rutin and ALI-related targets

The simplified molecular input line entry system (SMILES), the SDF 2D structure format, and the chemical structure of rutin were acquired from the PubChem database (https://pubchem.ncbi.nlm.nih.gov/). Then, the similarity ensemble approach (SEA) (http://sea.bkslab.org/) with max TC ≥ 50 (Keiser et al., 2007) and SwissTargetPrediction (http://www.swisstargetprediction.ch) with probability >0 as a condition for prediction (Daina et al., 2019) along with SuperPred (https://prediction.charite.de/index.php) (Nickel et al., 2014), and ChEMBL (https://www.ebi.ac.uk/chembl/) (Davies et al., 2015) databases were searched to find potential targets of rutin. Once duplication was removed, the identified targets were rectified using the Uniprot database (https://www.uniprot.org/), restricting the species to *Homo sapiens*.

Targets related to ALI were identified by entering acute lung injury as a keyword into three databases, GeneCards (https://www.genecards.org) with relevance score ≥10 (Stelzer et al., 2016), DisGeNET (https://www.disgenet.org/) (Piñero et al., 2017) with score ≥0.05 and protein-coding genes used as filtering conditions, and National Center for Biotechnology Information (NCBI) (https://www.ncbi.nlm.nih.gov/) gene function module.

To acquire the common genes, a Venn diagram was used to present the interaction between rutin targets and ALI-related genes.

### 2.2 Enrichment analyses

To enrich the functions of the intersected genes, the database for annotation, visualization, and integrated discovery (DAVID; v6.8, https://david.ncifcrf.gov/) was used to perform gene ontology (GO) analysis in terms of biological process (BP), cellular component (CC), and molecular function (MF), as well as the Kyoto encyclopedia of genes and genomes (KEGG) pathway analyses (Huang et al., 2009). Significant statistical difference was defined as FDR-adjusted *p* < 0.05.

### 2.3 Establishment of protein-protein interaction network and identification of hub genes

Exploring the interaction among the obtained shared genes was carried out by constructing a protein-protein interaction (PPI) network using the search tool for the retrieval of interacting genes (STRING; v12, http://string-db.org/), with limiting the target proteins to *Homo sapiens* and setting the minimum interaction score to medium confidence (0.400).

Cytoscape software (v3.10.1, www.cytoscape.org/) was subsequently used to visualize the PPI, and the topological properties of network nodes were analyzed using the Cytoscape Network Analyzer tool. The node size and color were set to be proportional to the node connectivity degree to promote the visual identification of the network. Additionally, the molecular complex detection (MCODE; v2.0.3, http://apps.cytoscape.org/apps/mcode) plug-in was used to select the biologically relevant subsets of network-related genes from the entire set. Furthermore, the CytoHubba plug-in (v0.1, http://hub.iis.sinica.edu.tw/cytohubba/) was applied to the subset with the highest MCODE score to select the top 10 hub genes according to several topological algorithms, including but not limited to edge percolated component (EPC), maximal clique centrality (MCC), and centralities based on shortest paths, such as Bottleneck (BN), and EcCentricity.

### 2.4 Prediction of upstream key miRNAs

Several online databases, including TargetScanHuman Release 8 (https://www.targetscan.org/vert_80/), miRTarBase (https://mirtarbase.cuhk.edu.cn), and DIANA-microT 2023 (https://dianalab.e-ce.uth.gr/microt_webserver/#/) (McGeary et al., 2019; Huang et al., 2022; Tastsoglou et al., 2023), were used to predict human and rat miRNAs that interact with the selected hub genes at the 3′untranslated region (3′UTR) of RNA transcripts. The identified miRNAs were then filtered to choose those in common between humans and rats. The miRNA/mRNA networks were visualized using Cytoscape.

Further, prediction of SM-miRNA regulation pairs by random forest (PSRR; https://rnadrug.shinyapps.io/PSRR/) (Yu et al., 2022) was applied to examine the impact of rutin on the shared miRNAs and identify a candidate miRNA for further study. To examine whether the putative miRNA can be translated from rats to humans, the conservation of the candidate miRNA was analyzed based on sequence annotations from miRBase (https://mirbase.org/). Further, RNAhybrid v2.2 (https://bibiserv.cebitec.uni-bielefeld.de/rnahybrid/) (Rehmsmeier et al., 2004) was used to analyze the formation of miRNA:mRNA duplexes with setting minimum free energy (ΔG) <−20 kcal/mol as the threshold.

### 2.5 *In vivo* experimental validation of the computational analyses

#### 2.5.1 Chemicals

Rutin was obtained from ThermoFisher Scientific (MA, USA), BLM was purchased as BLEOCEL^®^15 lyophilized powder containing bleomycin sulphate equivalent to 15 Units of bleomycin (Celon Labs, Telangana, India), and Bradford reagent was purchased from Sigma-Aldrich Co (MO, USA). All other chemicals and solvents were of the highest purity available.

#### 2.5.2 Animals

Thirty adult male Albino Wistar rats aged 8–10 weeks were obtained from the National Research Centre (NRC), Cairo, Egypt. Rats were housed in a well-ventilated room under standard conditions of temperature (26°C ± 2°C) and humidity (60% ± 5%) with 12/12 h light/dark cycles. Rats had access to a standard diet and water *ad libitum* and were left for acclimatization for 1 week before experimentation.

#### 2.5.3 Experimental design

Rats were randomly assigned into five groups consisting of six animals each, as follows: Group I served as the negative control group, Group II (rutin control group) administrated rutin dissolved in saline *per* oral route (p.o) using an intragastric tube at a dose of 200 mg/kg BW daily for 10 consecutive days (Liu et al., 2013; Zhang et al., 2023), Group III (BLM-induced group) anesthetized with an intravenous injection of a light sodium pentobarbital dose (50 mg/kg BW) and then received a single intratracheal instillation of BLM dissolved in saline at a dose of 5 mg/kg BW on the first day of the experiment. After instillation, rats were placed in a vertical position and spun for 1 min to ensure that the solution was distributed evenly within the lungs (Zhao et al., 2008; Guo et al., 2013), Group IV (rutin100 + BLM group) administrated rutin dissolved in saline p.o. at a low dose of 100 mg/kg BW/day for 10 days, and Group V (rutin200 + BLM group) administrated rutin dissolved in saline p.o. at a high dose of 200 mg/kg BW/day for 10 days. Animals in groups IV and V received a single intratracheal instillation of BLM at a dose of 5 mg/kg BW under anesthesia 2 h after rutin administration on day 1 of the study.

#### 2.5.4 Blood, bronchoalveolar lavage, and tissue sampling

At the end of the treatment period, rats were weighed and then fasted overnight. 24 h after the last dose of rutin, animals were anesthetized with an intraperitoneal injection of a Ketamine/Xylazine mixture at a dose of 100 mg/kg-10 mg/kg BW, followed by blood samples collection from the retro-orbital plexus into ethylene diamine tetra acetic acid (EDTA) coated tubes for hematological analysis.

Immediately after sampling, animals were sacrificed by cervical dislocation, and bronchoalveolar lavage was performed. Briefly, the trachea was cannulated, and the lungs were lavaged three times with 0.8 mL of 0.9% saline solution to collect bronchoalveolar lavage fluid (BALF).

Next, thoracosternotomy was carried out to excise the lungs, which were rinsed with saline, dried with filter papers, and weighed. Then, each side was divided into two parts; one for histopathological investigations, and the other was homogenized in ice-cold phosphate-buffered saline (PBS) to form 10% homogenate for biochemical assays. For molecular analyses, a small part of each side was snap-frozen in liquid nitrogen and stored at −80°C until used.

#### 2.5.5 Complete blood count

The collected peripheral blood was analyzed by the automated hematology analyzer XN-1000V (Sysmex, Hamburg, Germany) for complete blood count (CBC).

#### 2.5.6 Evaluation of lung index

To assess lung edema, the lung index was calculated based on lung weight and body weight as follows:

$$\text{Lung index }\left(\%\right)=\left[\text{lung weight }\left(g\right)/\text{body weight }\left(g\right)\right]\times 100$$

#### 2.5.7 Bronchoalveolar lavage analysis

After collection, BALF was centrifuged at 3,000 rpm for 10 min at 4°C. BALF total protein level was measured in the supernatant using bicinchoninic acid (BCA) assay kit, while monocyte chemoattractant protein-1 (MCP-1) and macrophage inflammatory protein-2 (MIP-2) levels were measured using commercial enzyme-linked immunosorbent assay (ELISA) kits (Cat# CSB-E07429r and CSB-E07419r, respectively) according to the manufacturer’s protocol (Cusabio).

The cell pellet was resuspended in PBS, and then total and differential cell counts were performed on a hemocytometer using Wright-Giemsa staining. Different cell types were determined based on the standard morphological profiles, with counting at least 300 cells/slide.

#### 2.5.8 Histopathology

The histological preparation technique was performed as reported by Bancroft and Gamble (Bancroft and Gamble, 2007). Concisely, lung tissues from the sacrificed animals were cut into 3–4 mm thick sections, fixed in 10% neutral buffered formalin, dehydrated in graded concentrations of ethanol, cleared in xylene, and embedded in paraffin. To study the general tissue structure, paraffin blocks were partitioned into sections using a microtome at a thickness of 4–6 μm and stained with Hematoxylin and Eosin (H and E) stain. Stained sections were examined using a Leica microscope (Leica Microsystems).

#### 2.5.9 Molecular analysis

To analyze the expression of rno-miR-9a-5p, miRNeasy Mini Kit (Qiagen) was used to extract total RNA from 30 mg lung tissue samples. After, reverse transcription was conducted using TaqMan™ MicroRNA Reverse Transcription Kit, and TaqMan™ MicroRNA Assay was used to conduct quantitative real-time PCR reactions (Applied Biosystems). The expression level of the mature miRNA was normalized to U6 snRNA. The PCR reactions included 1 μL of cDNA as a template, 10 μL of 2x TaqMan^®^ Universal PCR Master Mix (Applied Biosystems), 1 μL of 20x TaqMan™ MicroRNA Assay containing the primers and probe for the gene of interest as part of the kit (Cat# 4427975, assay ID: 000583 for rno-miR-9a-5p and 001,973 for U6 snRNA, Applied Biosystems), the volume was then completed to 20 μL with nuclease-free water.

For mRNA expression analysis, total RNA was extracted from lung tissues using the RNeasy Mini Kit according to the manufacturer’s instructions (Qiagen, Hilden, Germany). The High-Capacity cDNA Reverse Transcription kit and the TaqMan^®^ gene expression assay (Applied Biosystems) were used to perform the reverse transcription and quantitative real-time PCR, respectively, with the use of beta-actin (*Actb*) as the reference gene. The PCR reactions included 4 μL of cDNA as a template, 10 μL of 2x TaqMan^®^ universal PCR master mix (Applied Biosystems), 1 μL of 20x TaqMan^®^ gene expression assay which contained the primers and probe for the gene of interest as part of the kit (Cat# 4331182; assay ID: Rn01399572_m1 for *Nfkb1*, Rn01483828_m1for *Ptgs2*, Rn01483988_g1 for *Il10*, Rn01422083_m1 for *Il18*, Rn00594078_m1 for *Ifng*, and Rn00667869_m1for *Actb*, respectively) (Applied Biosystems), the volume was then completed to 20 μL with nuclease-free water.

In general, PCR reactions were conducted in the 7500 Real-Time PCR System (Applied Biosystems, CA, USA). The PCR thermal profile was 10 min at 95°C for enzyme activation, followed by 40 amplification cycles of denaturation at 95°C for 15 s and 60 s at 60°C for annealing and extension. Eventually, the 2^−ΔΔCt^ method was adopted for determining the quantitative measurements (Liva and Schmittgen, 2001).

#### 2.5.10 Immunohistochemical analysis

Immunohistochemical (IHC) staining was applied on paraffin tissue sections fixed on positively charged slides using rabbit polyclonal anti-nuclear factor-kappa-B (NF-κB) p50 and cyclooxygenase-2 (COX-2/prostaglandin-endoperoxide synthase 2) antibodies (Cat# E-AB-32226 and E-AB-62884) purchased from Elabscience (TX, USA); and all the reactions were developed using VECTASTAIN^®^ Elite^®^ ABC-HRP Kit (Cat# PK-6200, Vector laboratories) based on the avidin-biotin-peroxidase complex (ABC) method.

Firstly, paraffin sections were deparaffinized and hydrated through xylene and graded alcohol series. A subsequent quenching of endogenous peroxidase activity was carried out by incubating the slides in BLOXALL^®^ Blocking Solution (Cat# SP-6000, Vector laboratories, CA, USA) for 10 min. Sections were then incubated with Normal Serum reagent for 20 min to prevent non-specific background staining, followed by 30 min incubation with the anti-NF-κB p50 and anti-COX-2 antibodies (dilution 1:100). Slides were washed with PBS then incubated for 30 min with Biotinylated Universal Antibody. After washing with PBS, slides were incubated with VECTASTAIN Elite ABC reagent for 30 min and then washed with PBS. Markers' expression was detected with peroxidase and stained with DAB to recognize the antigen-antibody complex. Negative controls were integrated using non-immune serum instead of the primary or secondary antibodies.

Immuno-stained sections were checked and photographed using a Leica microscope (Leica Microsystems) under different magnification powers. Six high-power fields (x 400) exhibiting positive brown immunostaining were selected for evaluation in each serial section of the studied groups. Area (%) was determined for NF-κB p50 and COX-2 stained sections using a Leica QWin 500 image analyzer computer system (Leica Microsystems, Switzerland).

#### 2.5.11 Determination of pulmonary levels of IL-10, IL-18, and IFN-γ

Interleukin-10 (IL-10), IL-18, and interferon-gamma (IFN-γ) levels were determined in lung homogenates using commercial ELISA kits (Cat# CSB-E04595r, CSB-E04610r, and CSB-E04579r; Cusabio, TX, USA). The results were expressed as pg/mg protein after determining the protein concentration in lung homogenates using the Bradford method.

### 2.6 Statistical analysis

Statistical analysis was done using SPSS version 20.0 (IBM Corp, NY, USA). First, the Shapiro-Wilk test was used to verify the assumption of normal distribution of data. Normally distributed data were expressed as mean ± standard error of the mean (SEM) while non-normally distributed data were expressed as median and interquartile range (25th and 75th percentile). The statistical significance of differences among the studied groups was measured using one-way analysis of variance (ANOVA) followed by Tukey’s *post hoc* test for multiple comparisons or Kruskal-Walli’s test followed by Dunn’s *post hoc* for multiple comparisons as appropriate. A *p* < 0.05 was regarded as statistically significant.

## 3 Results

### 3.1 Target identification

**FIGURE 1:**
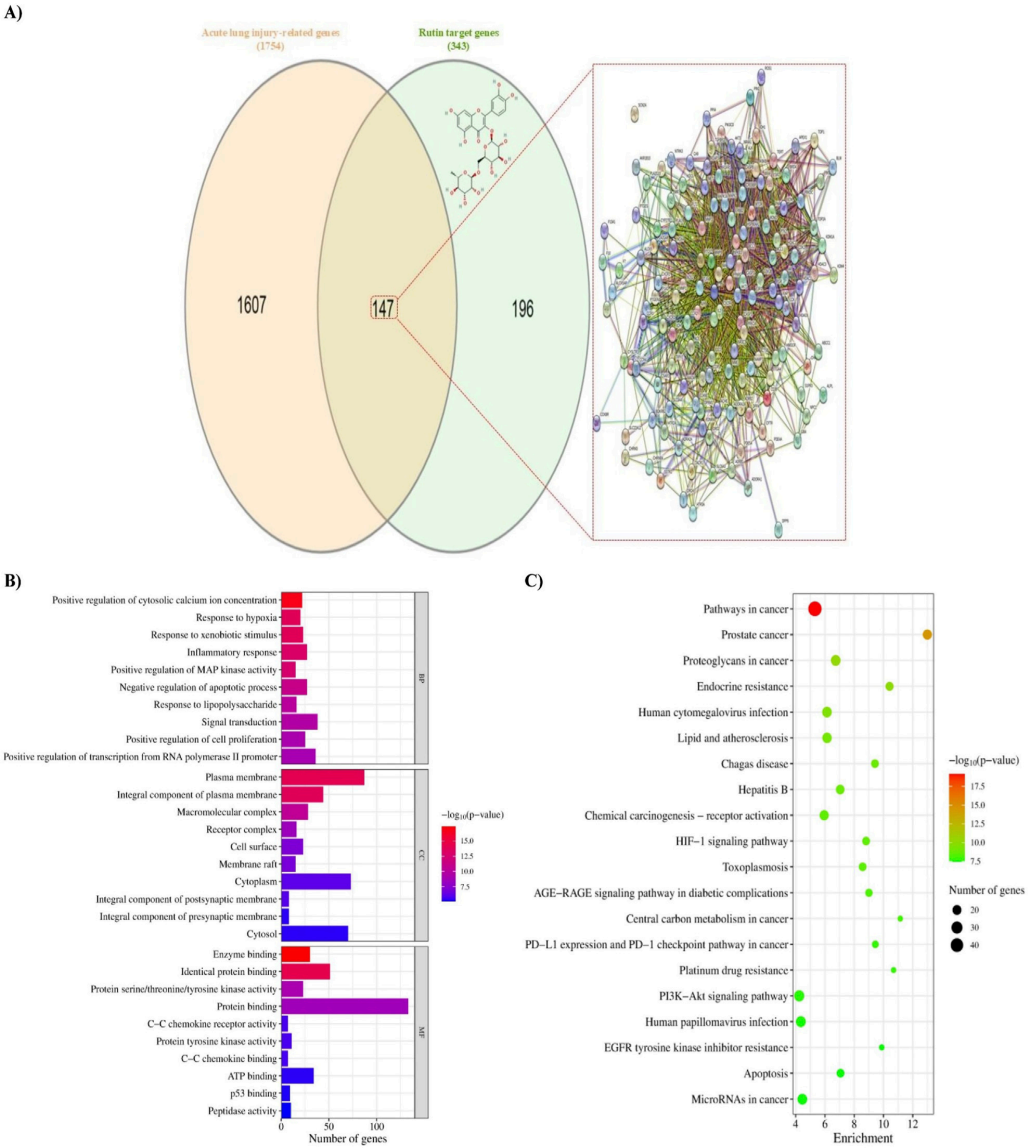
Identification of potential targets of rutin against BLM-induced acute lung injury and enrichment analyses. **(A)** Venn diagram of rutin targets alongside the 2D chemical structure of rutin, the acute lung injury-related genes, and the protein-protein interaction (PPI) network established from the diagram. Nodes represent proteins, and edges represent interactions. **(B)** Bar chart of GO terms analysis showing the top 10 terms in each of biological process (BP), cellular component (CC), and molecular function (MF). The color of the bars denotes the FDR-adjusted *p*-value, and the length represents the number of genes enriched in the term. **(C)** A Bubble chart of the KEGG pathway enrichment analysis showing the top 20 putative signaling pathways according to the FDR-adjusted *p*-value. The color of the circles represents the *p*-value, and the size represents the number of genes enriched in the pathway. The direction towards red color indicates more significance. BLM: Bleomycin.

After eliminating redundancy, 343 unique targets of rutin were obtained from SEA, SwissTargetPrediction, SuperPred, and ChEMBL databases. Additionally, 1754 ALI-related genes were identified from the GeneCards, DisGeNET, and NCBI databases (Supplementary Files S1, S2). The targets were then mapped *via* a Venn diagram, yielding 147 intersected genes that were considered, in turn, potential targets of rutin action against ALI (Figure 1A).

### 3.2 Functional enrichment analysis

GO and KEGG enrichment analyses were conducted on the intersecting targets to clarify the fundamental mechanism of rutin action. Of 602 BP, 78 CC, and 127 MF terms identified, only 255, 49, and 57 were statistically significant, respectively (Supplementary File S3). According to the *p*-value, the top five terms in the BP category were positive regulation of cytosolic calcium ion concentration, response to hypoxia, response to xenobiotic stimulus, inflammatory response, and positive regulation of MAP kinase activity. The strongly connected MFs with these biological processes were enzyme binding, identical protein binding, protein serine/threonine/tyrosine kinase activity, protein binding, and protein tyrosine kinase activity. Further, these processes occurred mainly in plasma membrane, integral component of plasma membrane, macromolecular complex, receptor complex, and cell surface. The top 10 enriched terms in each category according to the *p*-value were selected for visualization (Figure 1B).

Moreover, the results of KEGG enrichment analysis demonstrated that these targets are enriched in 164 pathways, of which 153 pathways are statistically significant, mainly involving pathways in cancer, prostate cancer, proteoglycans in cancer, endocrine resistance, human cytomegalovirus infection, lipid and atherosclerosis, Chagas disease, hepatitis B, chemical carcinogenesis - receptor activation, and hypoxia-inducible factor-1 (HIF-1) signaling pathway. The top 20 pathways according to the *p*-value are represented in Figure 1C.

### 3.3 PPI network analysis

**FIGURE 2:**
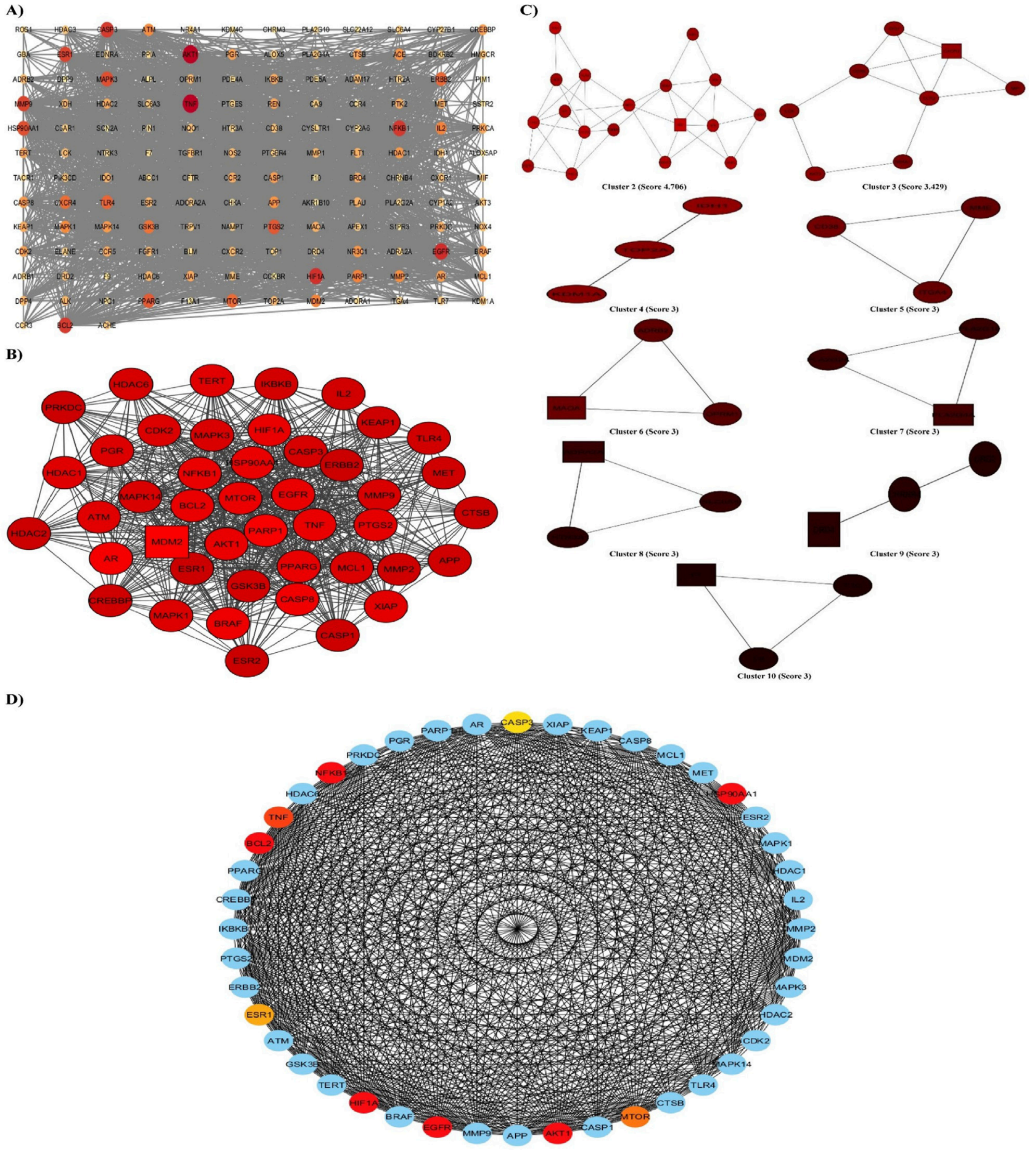
PPI network and screening of the hub genes. **(A)** STRING database was used to analyze the potential targets, giving a network composed of 147 nodes and 2017 edges, which was then displayed on Cytoscape. The node size and color reflect the node degree, and the edges represent the interactions between nodes. **(B, C)** PPI network based on clustery analysis using the MCODE plug-in produced 10 modules, the top with a score of 33.86, 44 nodes, and 728 edges. The node color is according to the MCODE score, and the node shape is according to the node status; circles represent clustered nodes, and the rectangle represents the seed node. **(D)** The top 10 hub genes were identified in cluster one using the CytoHubba plug-in. The node’s color represents its score according to the maximal clique centrality (MCC) algorithm; the darker the color, the higher the score.

The PPI network was constructed based on the 147 putative targets of rutin thought to ameliorate ALI. The network was visualized and analyzed using Cytoscape software. As presented in Figure 2A, the network contains 147 nodes and 2017 edges with an average node degree of 27.4, an average local clustering coefficient of 0.592, and a PPI enrichment *p*-value < 1.0e-16. The characteristic path length between all node pairs was 1.983, whereas the network diameter, radius, density, and heterogeneity were 4, 2, 0.191, and 0.786, respectively. The topological features of each node, including but not limited to Degree, Betweenness centrality, and Closeness centrality, were analyzed by the Cytoscape Network analyzer tool (Supplementary File S4). The node size and color in the network were positively related to the node degree. When the targets were sorted by degree, *TNF*, *AKT1*, *EGFR*, *HIF1A*, and *NFKB1* were identified as the five core targets.

Moreover, the network was further assessed with the MCODE plug-in to detect modules/clusters (densely connected regions), suggesting functional protein complexes. It revealed 10 functional modules of clustered genes sorted by their score (Figures 2B, C). The cluster with the highest score (33.86) comprised 44 nodes and 728 edges. Consequently, the top 10 hub genes in this cluster were recognized using the different topological algorithms and centralities based on shortest paths of the CytoHubba plug-in.

Figure 2D shows the ranking of hub genes according to the MCC algorithm. Although *NFKB1*, *AKT1*, *HIF1A*, *EGFR*, *BCL2*, and *HSP90AA1* genes had the highest and the same score among the genes of cluster 1, the *NFKB1* gene appears to be the most important according to the sum of the other parameters.

The node status in the network can be revealed from different facets by parameters calculated using multiple algorithms. Hence, the results of the three methods (the topological features analysis, MCODE, and CytoHubba) established a solid basis for selecting the hub genes for further experimental verification. Among the target genes, *NFKB1* appears to be the most notable.

### 3.4 Prediction of the upstream miRNAs

**FIGURE 3:**
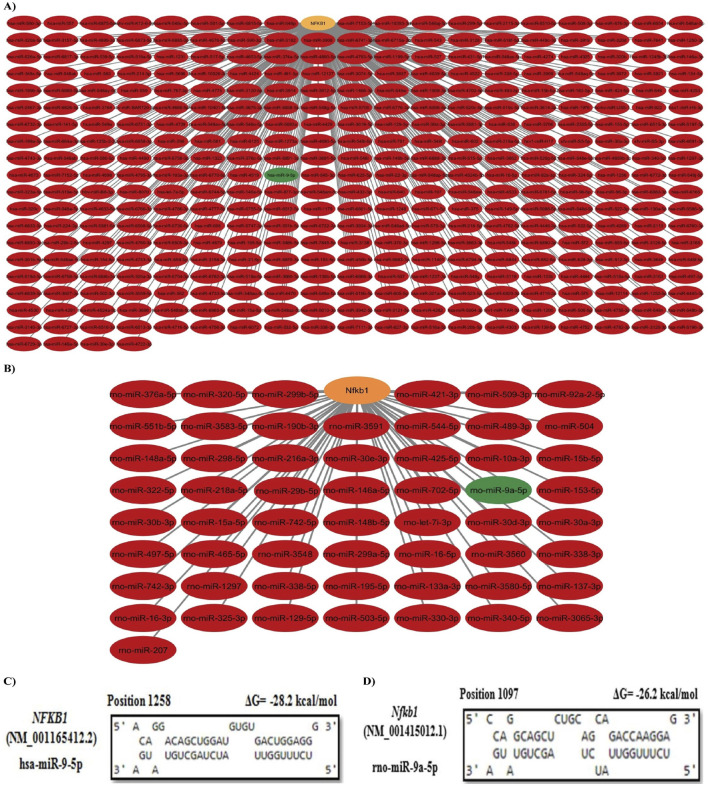
Prediction and selection of the upstream key miRNA. **(A)** Human miRNAs/*NFKB1* interaction network. **(B)** Rat miRNAs/*Nfkb1* interaction network. The orange node represents the target gene, and the green nodes represent shared miRNAs between humans and rats. **(C, D)** miRNA:mRNA duplex formation analysis. Hsa-miR-9-5p was predicted to target *NFKB1* at position 1258 with ΔG = −28.2 kcal/mol, whereas rno-miR-9a-5p was predicted to target *Nfkb1* at position 1097 with ΔG = −26.2 kcal/mol.

TargetScanHuman Release 8, miRTarBase, and DIANA-microT 2023 databases were used to predict the upstream miRNAs that target the *NFKB1* gene in humans and rats. After merging and removing duplicates, 364 miRNAs were found to target *NFKB1* in humans, while only 56 were identified in rats. Among them, the shared miRNAs were hsa-miR-9-5p and rno-miR-9a-5p (Supplementary File S5, Figures 3A, B). Additionally, the PSRR web server predicted that rutin is able to upregulate the expression of hsa-miR-9-5p with a rate of 0.79.

According to the miRBase database, the sequence of rno-miR-9a-5p, UCU​UUG​GUU​AUC​UAG​CUG​UAU​GA, was 100% identical to that of hsa-miR-9-5p. Therefore, it was considered a conserved candidate miRNA. To assess the interaction between miR-9-5p and *NFKB1*, miRNA:mRNA duplex formation was analyzed using RNAhybrid. Both hsa-miR-9-5p and rno-miR-9a-5p were found to interact with *NFKB1* and *Nfkb1* mRNAs at multiple sites. Figures 3C, D show the targeting site of hsa-miR-9-5p (at position 1258) and rno-miR-9a-5p (at position 1097) with the highest number of continuous base pairing and the lowest binding energy (ΔG = −28.2 kcal/mol and −26.2 kcal/mol, respectively) indicating high binding strength in miRNA:mRNA duplex formation.

### 3.5 Effect of rutin on CBC

**FIGURE 4:**
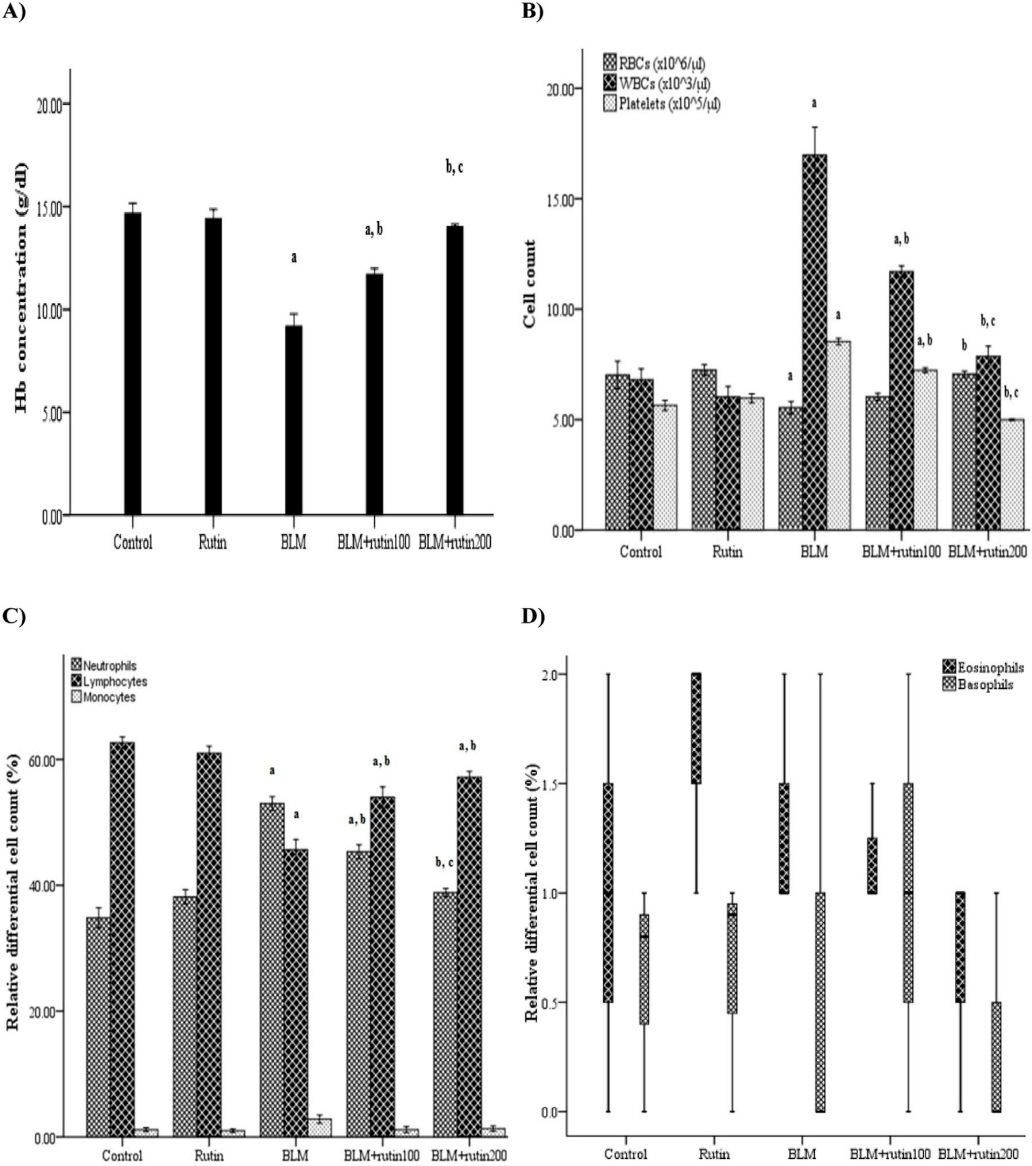
Effect of rutin on some CBC parameters. **(A)** Hb concentration, **(B)** RBCs, WBCs, and platelets count and **(C, D)** WBCs differential count. CBC, Complete blood count; BLM, Bleomycin; rutin100, Rutin at a dose of 100 mg; rutin200, Rutin at a dose of 200 mg; Hb, Hemoglobin; RBCs, Red blood corpuscles; WBCs, White blood cells. In multiple comparisons, ^a^ *p* < 0.05 vs. normal control group, ^b^ *p* < 0.05 vs. BLM-induced group, and ^c^ *p* < 0.05 vs. BLM + rutin100 group. n = 6 for each experimental group. Bar length represents the mean value for each group, with error bars depicting standard error of mean. Box plot indicates median and interquartile range.

Figure 4 demonstrates a non-significant change in the CBC parameters between rutin control group and the negative control group. On the other hand, the BLM-induced group showed a significant decrease in hemoglobin (Hb) concentration (*p* < 0.001) and red blood corpuscles (RBCs) count (*p* = 0.032), accompanied by a dramatic increase in platelet and white blood cells (WBCs) count (*p* < 0.001). Treatment with rutin, at doses of 100 mg and 200 mg/kg BW, mitigated the effect of BLM by inducing a significant increase in the Hb concentration (*p* = 0.003 and *p* < 0.001, respectively), whereas only the high dose induced a significant increase in the RBC count (*p* = 0.027). In addition, rutin treatment at both doses decreased the platelet count (*p* < 0.001) and WBC count (*p* = 0.005 and *p* < 0.001, respectively). The effect of the rutin high dose was more marked than the low dose regarding the Hb concentration (*p* = 0.006), platelet count (*p* < 0.001), and WBC count (*p* = 0.005) (Figures 4A, B).

For the differential WBC count, the relative neutrophil count was increased significantly with the decrease of the lymphocyte percentage in the BLM-induced group compared to the negative control group (*p* < 0.001). Rutin treatment reversed these alterations at both doses (*p* = 0.001 and *p* < 0.001, respectively). When considering the relative neutrophil count, the effect of the 200 mg dose was more pronounced than that of the 100 mg dose (*p* = 0.005). Further, all the studied groups showed a non-significant difference regarding monocyte, eosinophil, and basophil relative count (Figures 4C, D).

### 3.6 Effect of rutin on lung index, total protein level, and inflammatory cell infiltration in the BALF

**FIGURE 5:**
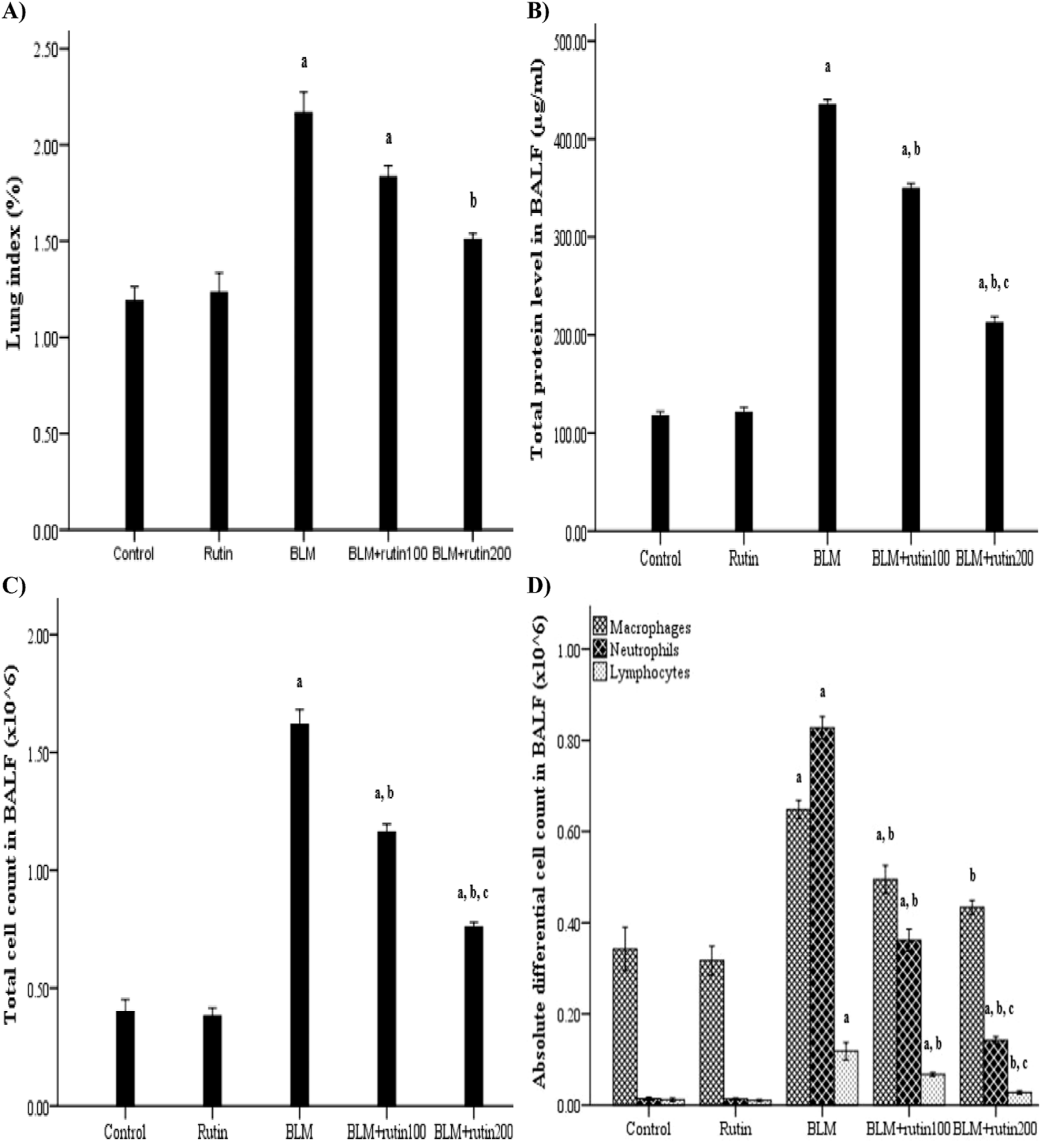
Rutin impact on **(A)** lung index, **(B)** total protein level in the BALF, **(C)** inflammatory cell infiltration in the BALF and **(D)** differential cell count in the BALF. BLM, Bleomycin; rutin100, Rutin at a dose of 100 mg; rutin200, Rutin at a dose of 200 mg; BALF, Bronchoalveolar lavage fluid. In multiple comparisons, ^a^ *p* < 0.05 vs. normal control group, ^b^ *p* < 0.05 vs. BLM-induced group, and ^c^ *p* < 0.05 vs. BLM + rutin100 group. n = 6 for each experimental group. Bar length represents the mean value for each group, with error bars depicting standard error of mean.

The lung index was significantly increased in the BLM-induced group compared to negative controls (2.17% ± 0.11% vs. 1.19% ± 0.07%, *p* < 0.001), indicating severe pulmonary edema. Although rutin treatment at a dose of 100 mg produced a borderline non-significant decrease in the lung index by 15.67% (*p* = 0.085), the treatment with the higher dose was able to reduce the lung index significantly by 30.41% (*p* = 0.001) compared to the BLM-intoxicated group (Figure 5A).

Regarding BALF total protein content, it did not vary significantly between negative control and rutin control groups. Meanwhile, BLM increased the capillary permeability of alveoli after lung injury, which led to protein leakage as evidenced by a significant rise in the total protein level in BALF compared to controls (434.81 ± 5.17 μg/mL vs. 117.61 ± 4.44 μg/mL, *p* < 0.001). By contrast, this increase was significantly inhibited by rutin administration at both doses by 19.63% and 51.17%, respectively (*p* < 0.001) (Figure 5B).

Figure 5C illustrates the significant increase in the total cell count in the BALF of BLM-exposed animals compared to controls (1.62 ± 0.06 × 10^6^ cell vs. 0.40 ± 0.05 × 10^6^ cell, *p* < 0.001). However, rutin treatment reversed this increase by 28.39% and 53.09% (*p* < 0.001) at doses of 100 mg and 200 mg, respectively.

In the same context, the macrophage, neutrophil, and lymphocyte counting were significantly higher in the BLM-induced group than in the negative control group (0.65 ± 0.02 × 10^6^ cell vs. 0.34 ± 0.05 × 10^6^ cell, 0.83 ± 0.02 × 10^6^ cell vs. 0.01 ± 0.003 × 10^6^ cell, and 0.12 ± 0.02 × 10^6^ cell vs. 0.01 ± 0.004 × 10^6^ cell, *p* < 0.001, respectively), demonstrating inflammatory cell migration to the injured lungs. Rutin at a dose of 200 mg was able to inhibit inflammatory cell recruitment by 33.85%, 83.13%, and 76.67% (*p* < 0.001), respectively. On the other hand, rutin at a dose of 100 mg was able to decrease the inflammatory cell infiltration by 24.61% (*p* = 0.015), 56.63% (*p* < 0.001), and 43.33% (*p* = 0.004), respectively. Despite rutin administration at the high dose produced a more pronounced mitigated effect than the lower dose regarding neutrophil and lymphocyte count (*p* < 0.001 and *p* = 0.032, respectively), a non-significant difference was observed when considering macrophage count (*p* = 0.641) (Figure 5D).

### 3.7 Effect of rutin on MCP-1 and MIP-2 production level

As shown in Table 1, MCP-1 and MIP-2 levels in BALF were comparable between the negative control and rutin control groups (*p* = 0.963 and *p* = 0.321, respectively). Meantime, the BLM injection significantly increased the production level of these inflammatory mediators by 5.25 and 2.89 folds, respectively, compared to the control group (*p* < 0.001). Nonetheless, their levels were markedly decreased upon rutin administration by 37.15% and 20.75% (*p* < 0.001) at a dose of 100 mg and by 70% and 50.29% (*p* < 0.001) at a dose of 200 mg, respectively. Once more, the high dose of rutin was more efficacious than the lower in alleviating cytokine production in BALF (*p* < 0.001).

**TABLE 1:** Effect of rutin on MCP-1 and MIP-2 levels in BALF.

	MCP-1 (pg/mL)	MIP-2 (pg/mL)
Control	60.03 ± 3.11	42.20 ± 1.66
Rutin	63.08 ± 4.02	47.07 ± 1.33
BLM	375.50 ± 2.95^a^	164.01 ± 2.07^a^
BLM + rutin100	236.00 ± 3.38^a^ ^,^ ^b^	129.97 ± 1.52^a^ ^,^ ^b^
BLM + rutin200	112.69 ± 2.76^a^ ^,^ ^b^ ^,^ ^c^	81.52 ± 2.14^a^ ^,^ ^b^ ^,^ ^c^

Data are expressed as mean ± SEM. n = 6 for each experimental group. MCP-1: Monocyte chemoattractant protein-1, MIP-2: Macrophage inflammatory protein-2, BALF: bronchoalveolar lavage fluid, BLM: bleomycin, rutin100: Rutin at a dose of 100 mg, rutin200: Rutin at a dose of 200 mg.

In multiple comparisons,

^a^ *p* < 0.05 vs. normal control group.

^b^ *p* < 0.05 vs. BLM-induced group, and.

^c^ *p* < 0.05 vs. BLM + rutin100 group.

### 3.8 Histopathological examination

**FIGURE 6:**
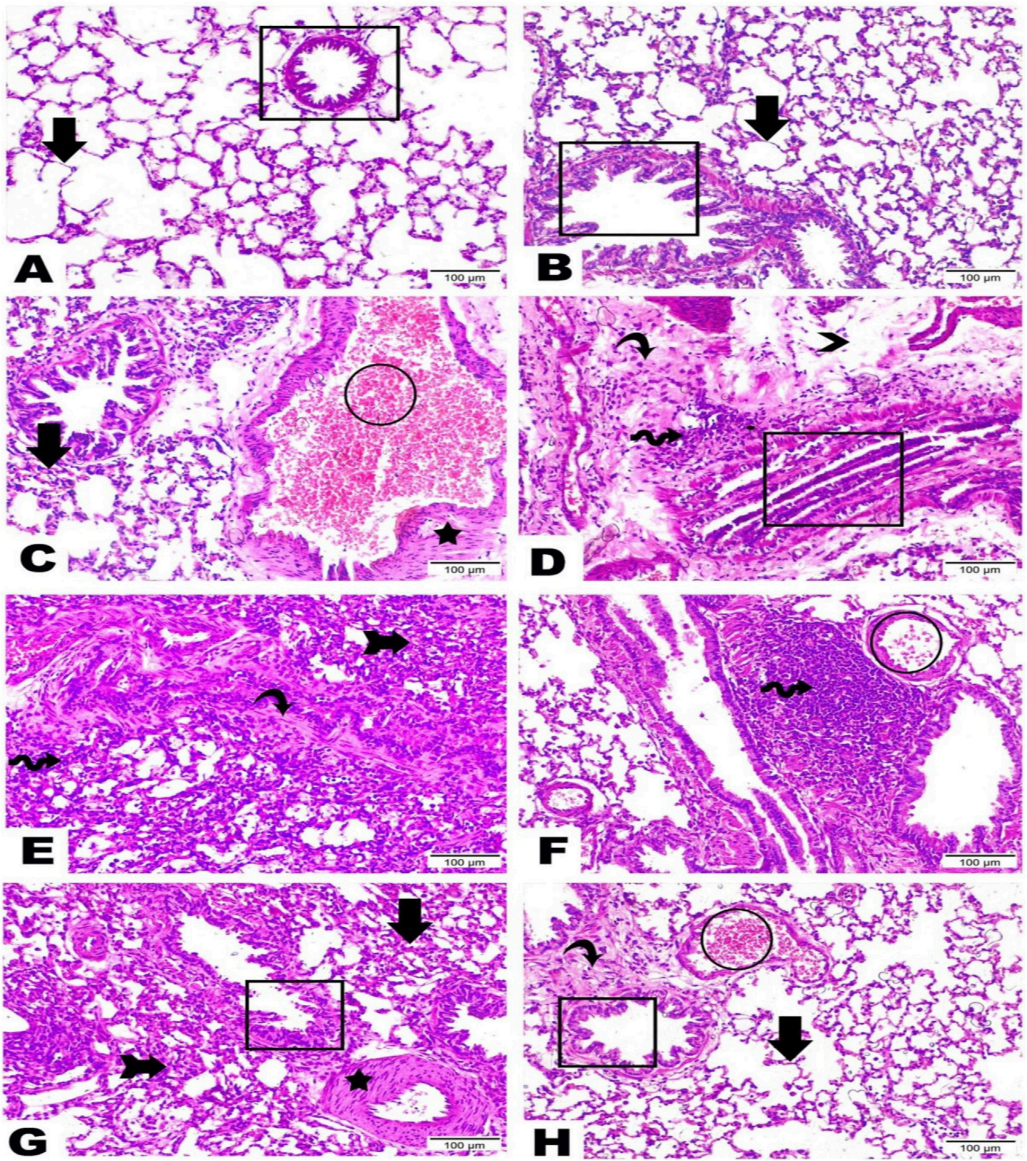
Representative photomicrographs demonstrating the histopathological alterations in lung tissue sections among examined groups (H and E, x200). Lung sections from **(A)** Negative control group, **(B)** Rutin control group, **(C–E)** Bleomycin-induced group, **(F, G)** BLM + rutin100 group, and **(H)** BLM + rutin200 group highlighted the changes between groups along bronchioles (rectangle), alveolar epithelium (arrows), loss of lung architecture (arrow with tail), blood vessels (circle), smooth muscle (star), inflammatory cells (wave arrow), interstitial edema (arrowhead), fibrous connective tissue (curvy arrow). BLM, Bleomycin; rutin100, Rutin at a dose of 100 mg; rutin200, Rutin at a dose of 200 mg.

Lung sections from negative control and rutin control groups denoted the standard architecture of lung tissue assembled in normal bronchioles and intact lung alveoli (Figures 6A, B). Otherwise, the BLM-induced group exhibited serious degenerative changes with loss of lung architecture evidenced by obvious congestion and dilatation along blood vessels, thickening in alveolar epithelium and smooth muscle, massive number of inflammatory cells infiltration, interstitial edema, hyperplasia of bronchiole lining epithelium, as well as noticeable increase in fibrous amount (Figures 6C–E). Treatment with rutin at a dose of 100 mg produced moderate improvements noticed by the existence of a high quantity of inflammatory cells, few congestions along blood vessels, a great reduction of bronchiole hyperplasia lining epithelium, as well as the thickening of muscle. Furthermore, some alveolar epithelium was noticed in the regular form while others looked with loss architecture (Figures 6F, G). Whereas the high rutin dose (200 mg) led to a better enhancement that was distinguished by the presence of a scarce number of inflammatory cells, less dilated and congested blood vessels, besides intact existence of bronchiole lining epithelium as well as alveolar epithelium (Figure 6H).

### 3.9 Alteration of the pulmonary expression level of rno-mir-9a-5p, *Nfkb1*, and some related genes in response to rutin

**FIGURE 7:**
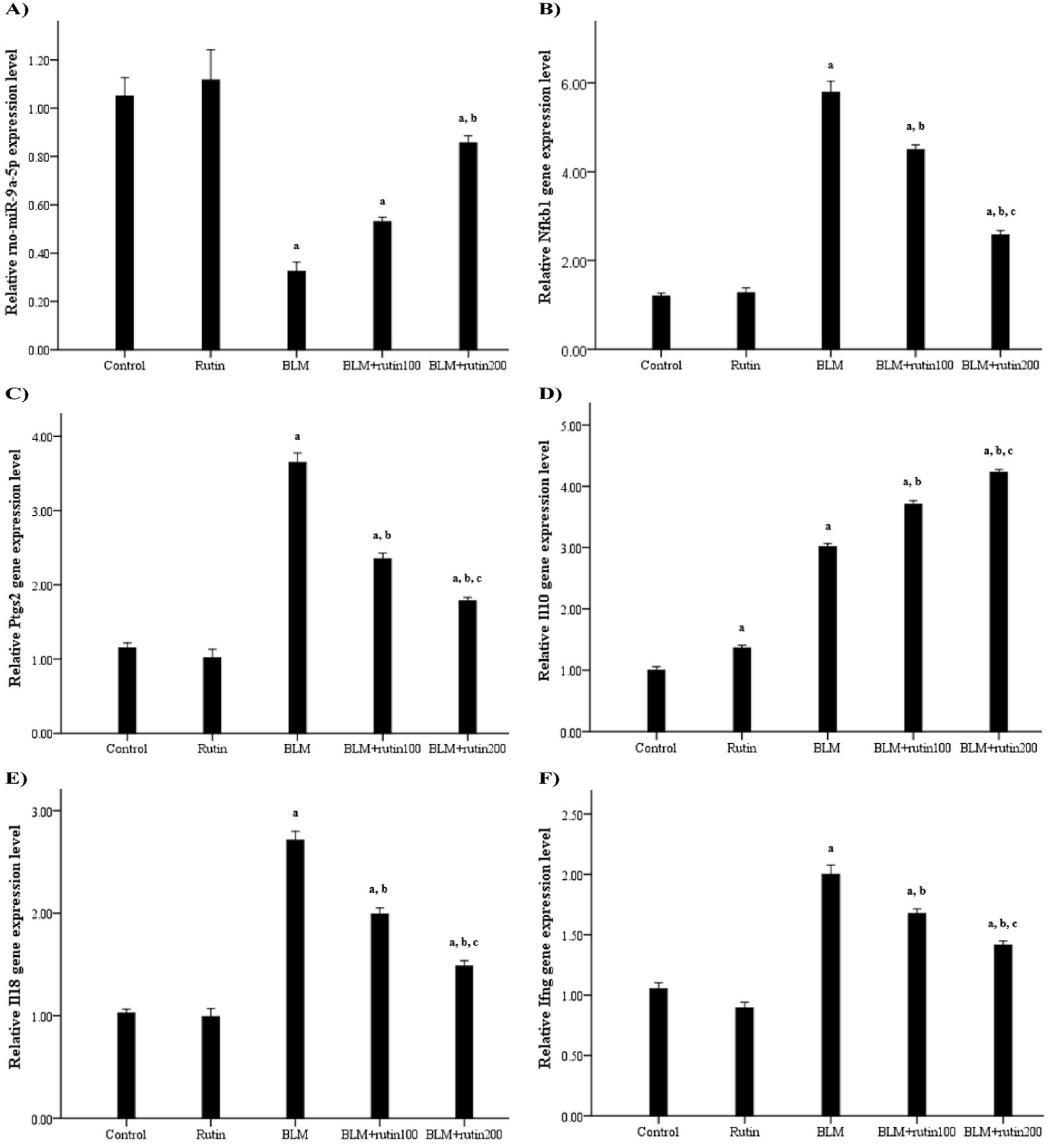
Effect of rutin on the pulmonary expression levels of **(A)** rno-miR-9a-5p, **(B)** *Nfkb1*, and some related downstream genes, including **(C)** *Ptgs2*, **(D)** *Il10*, **(E)** *Il18*, and **(F)** *Ifng*. BLM, Bleomycin; rutin100, Rutin at a dose of 100 mg; rutin200, Rutin at a dose of 200 mg; Nfkb1, Nuclear factor kappa B subunit 1; Ptgs2, Prostaglandin-endoperoxide synthase 2; Il10, Interleukin-10; Il18, Interleukin-18; Ifng, Interferon-gamma. In multiple comparisons, ^a^ *p* < 0.05 vs. normal control group, ^b^ *p* < 0.05 vs. BLM-induced group, and ^c^ *p* < 0.05 vs. BLM + rutin100 group. n = 6 for each experimental group. Bar length represents the mean value for each group, with error bars depicting standard error of mean.

Regarding rno-miR-9a-5p expression, a significant downregulation was observed in BLM-challenged animals by 69.52% compared to the negative control group (*p* < 0.001). In contrast, the administration of rutin lessened the impact of BLM, causing upregulation of the miRNA expression level by 65.62% and 1.69-fold at a dose of 100 mg and 200 mg, respectively (*p* < 0.001) (Figure 7A).

Concerning mRNA expression level, the BLM injection resulted in a significant upregulation of the pulmonary expression levels of *Nfkb1* and the pro-inflammatory genes *Ptgs2, Il18*, and *Ifng* (by 3.82, 2.17, 1.66, and 0.9 folds, respectively, *p* < 0.001) in comparison with negative control animals. On the contrary, rutin administration significantly ameliorated these effects at the low dose by 22.28% (*p* < 0.001), 35.62% (*p* < 0.001), 26.57% (*p* < 0.001), and 16.5% (*p* = 0.002), respectively, and the high dose (by 55.61%, 51.23%, 45.39%, and 29.00%, respectively, *p* < 0.001). For the anti-inflammatory *Il10* gene, rutin control group showed a significant upregulation by 37% compared to the negative control group (*p* < 0.001), reflecting rutin’s anti-inflammatory properties. Interestingly, BLM induced the expression of *Il10* by 2.01-folds compared to negative controls (*p* < 0.001), while rutin treatment led to a further robust increase by 20.27% and 40.53% at a dose of 100 mg and 200 mg, respectively (*p* < 0.001) compared to the BLM-intoxicated group (Figures 7B–F).

Notably, rutin administration produced a powerful effect at the high dose as regards the pulmonary expression levels of rno-miR-9a-5p, *Nfkb1*, *Ptgs2*, *Il18*, *Ifng*, and *Il10* than the low dose (*p* = 0.021, *p* < 0.001, *p* = 0.001, *p* < 0.001, *p* = 0.015, and *p* < 0.001, respectively).

### 3.10 Influence of rutin on the pulmonary expression level of NF-κB p50 and COX-2

**FIGURE 8:**
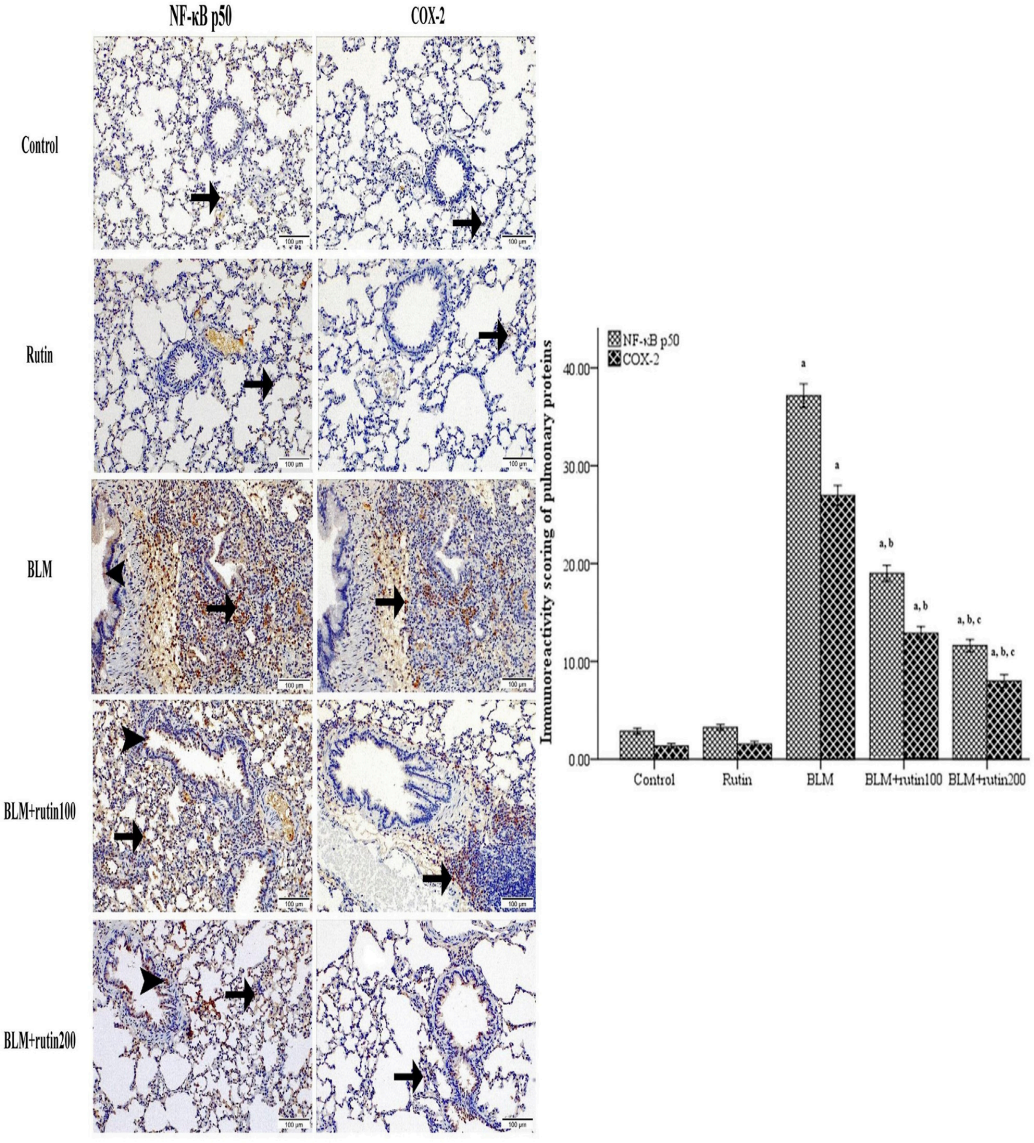
Pulmonary NF-κB p50 and COX-2 expression level in the studied groups using immunohistochemistry. Lung sections highlighted with positive cytoplasmic expressions along bronchiole epithelium (arrowhead) or/and interstitial tissue (arrow) with scarce reactivity along negative control and rutin control groups, highest in BLM-induced group, moderate in BLM + rutin100 group, and few in BLM + rutin200 group. The bar chart represents the immune scoring area (%) of NF-κB p50 and COX-2. NF-κB p50, Nuclear factor-kappa-B p50; COX-2, Cyclooxygenase-2; BLM, Bleomycin; rutin100, Rutin at a dose of 100 mg; rutin200, Rutin at a dose of 200 mg. In multiple comparisons, ^a^ *p* < 0.05 vs. normal control group, ^b^ *p* < 0.05 vs. BLM-induced group, and ^c^ *p* < 0.05 vs. BLM + rutin100 group. n = 6 for each experimental group. Bar length represents the mean value for each group, with error bars depicting standard error of mean.

IHC testing and quantitative scoring of NF-κB p50 and COX-2 along lung tissue sections between examined groups (Figure 8) unveiled that negative control and rutin control groups exhibited scarce positive cytoplasmic reactivity with non-significant differences. The BLM-induced group underscored the highest strong positive reactivity with a significant difference compared to the negative control group (*p* < 0.001). Although animals treated with rutin at a dose of 100 mg demonstrated moderate positive reactivity with a significant difference compared to the BLM-induced group (*p* < 0.001), animals treated with rutin at the high dose (200 mg) showed fewer positive expressions with a significant difference compared to BLM-induced group and animals treated with the lower rutin dose (*p* < 0.001).

### 3.11 Effect of rutin on the pulmonary IL-10, IL-18, and IFN-γ levels

Table 2 demonstrates that pulmonary levels of the pro-inflammatory cytokines, IL-18 and IFN-γ, were dramatically elevated in the BLM-induced group compared to the negative control group by 2.79 and 1.99-folds, respectively (*p* < 0.001). On the contrary, these levels were considerably decreased upon rutin administration at the low dose (by 32.10% and 25.85%, respectively, *p* < 0.001) and the high dose (by 53.81% and 44.75%, respectively, *p* < 0.001).

**TABLE 2:** Effect of rutin on some inflammatory markers in lung tissues.

	IL-10 (pg/mg protein)	IL-18 (pg/mg protein)	IFN-γ (pg/mg protein)
Control	110.40 ± 11.06	25.27 ± 1.40	24.12 ± 2.61
Rutin	228.38 ± 18.93^a^	22.64 ± 1.37	23.59 ± 2.24
BLM	457.21 ± 29.72^a^	95.88 ± 1.91^a^	72.25 ± 1.28^a^
BLM + rutin100	602.66 ± 28.14^a^ ^,^ ^b^	65.10 ± 1.93^a^ ^,^ ^b^	53.57 ± 1.22^a^ ^,^ ^b^
BLM + rutin200	813.15 ± 28.95^a^ ^,^ ^b^ ^,^ ^c^	44.29 ± 1.47^a^ ^,^ ^b^ ^,^ ^c^	39.92 ± 2.70^a^ ^,^ ^b^ ^,^ ^c^

Data are expressed as mean ± SEM. n = 6 for each experimental group. IL-10: Interleukin-10, IL-18: Interleukin 18, IFN-γ: Interferon-gamma, BLM: bleomycin, rutin100: Rutin at a dose of 100 mg, rutin200: Rutin at a dose of 200 mg.

In multiple comparisons,

^a^ *p* < 0.05 vs. normal control group.

^b^ *p* < 0.05 vs. BLM-induced group, and.

^c^ *p* < 0.05 vs. BLM + rutin100 group.

On the other hand, rutin administration alone caused a notable rise in the IL-10 pulmonary level by 1.07-fold (*p* = 0.017) compared to the negative control group. Additionally, BLM intoxication led to a significant increase in IL-10 pulmonary level by 3.14 folds (*p* < 0.001). Noteworthy, rutin treatment resulted in a further elevation in the pulmonary level of this anti-inflammatory cytokine by 31.81% (*p* = 0.002) at the low dose and 77.85% (*p* < 0.001) at the high dose compared to the BLM-induced group.

Further, rutin administration at a dose of 200 mg induced a significant decrease in IL-18 and IFN-γ levels (*p* < 0.001 and *p* = 0.001, respectively) and a marked increase in IL-10 level (*p* < 0.001) in lung tissues compared to the dose of 100 mg.

## 4 Discussion

Despite its benefits as a chemotherapeutic agent for numerous human malignancies, the well-known severe side effects of BLM, including ALI, seem inevitable in most patients (Shippee et al., 2016). Because of the poor prognosis, availability of supportive therapy only as the primary treatment, low response of patients, and high mortality rate of 30%–40% (Mokrá, 2020), there is an urgent need to develop new drugs to treat or delay the progress of ALI. Thus, the current study was designed to explore the potentiality of rutin against BLM-induced ALI.

It is well established that BLM binds with Fe^2+^, causing iron deficiency and anemia, thereby inducing oxidative stress in RBCs directly and/or indirectly by binding to the DNA, initiating membrane lipid peroxidation, and enhancing reactive oxygen species (ROS) production (Segerman et al., 2013; Allawzi et al., 2019). This is in line with the results of the present study that demonstrated a marked decrease in Hb concentration and RBC count in the BLM-induced group compared to the negative control group. Meanwhile, treatment with rutin mitigated the effect of BLM by inducing a significant increase in the Hb concentration at both doses and RBC count at the high dose only. This is likely due to the antioxidant property of rutin, quenching ROS generation in erythrocytes (López-Revuelta et al., 2006).

Rutin’s antioxidant capacity may be attributed to its remarkable potency as a reducing agent, hydrogen donor, and free radical quencher. Based on the structure-activity relationship, it is plausible that the four hydroxyl groups rutin possess could be responsible for this biological activity (Lue et al., 2010). Notably, this capacity could prevent the induction of inflammatory cytokine transcription factors, making rutin effective for treating inflammatory diseases (Koval’skii et al., 2014).

Platelets are implicated in a variety of inflammatory disorders, particularly respiratory conditions such as asthma and ALI, partly *via* promoting leukocyte recruitment to the lung microvasculature (Momi et al., 2017; Zarbock and Ley, 2008). However, there are contradictory results about the platelet count in BLM-induced lung injury. Gad and co-investigators (Gad El-Hak et al., 2022), on one hand, reported a significant reduction in platelet count in the BLM-induced group. On the other hand, El-Fakharany and colleagues (El-Fakharany et al., 2023) found a significant increase in the platelet count as a consequence of BLM intoxication, which is in agreement with the results of the current work that demonstrated a remarkable elevation of the peripheral blood platelets in the BLM-treated animals compared to negative controls.

Additionally, intratracheal BLM instillation increased total WBC count with the elevation of relative neutrophil count in the periphery. Further, total cell, macrophage, neutrophil, and lymphocyte counts were markedly increased in the BALF compared to negative controls. This is a hallmark of ALI, reflecting parenchymal injury and primary inflammatory lesions characterized by the accumulation of alveolar inflammatory cells in the lower respiratory tract (Pugin et al., 1999). Hence, uncontrollable inflammatory responses represented by increased the permeability of the alveolar/capillary barrier, leakage of intravascular plasma fluid, and pulmonary edema were observed in BLM-challenging animals, as evidenced by increased lung index and total protein level in BALF. These findings concur with the results of previous studies (Yu et al., 2016; Zaghloul et al., 2019; Kseibati et al., 2020).

Further, the infiltrated inflammatory cells secrete massive amounts of pro-inflammatory cytokines that act as chemoattractants to recruit more inflammatory cells, particularly neutrophils and macrophages. This amplifies the signal cascade and, ultimately, leads to lung injury (Deshmane et al., 2009; Dolinay et al., 2012). This aligns with the results of the present study that showed elevated MCP-1 and MIP-2 levels in the BALF of the BLM-induced group compared to the negative control group. MCP-1 is a pro-inflammatory protein produced by endothelial cells, alveolar epithelial cells, and macrophages. It belongs to the C-C chemokine family and regulates the migration and infiltration of monocytes/macrophages. Thus, it is implicated in the pathogeneses of various inflammatory diseases and has been reported to play a vital role in developing lung inflammation and fibrosis (Prasse and Müller-Quernheim, 2009; Gao et al., 2016). MIP-2 is another powerful chemoattractant released by a variety of cells. It belongs to the C-X-C chemokines and contributes to initiating and extending the inflammatory process through chemical chemotaxis and activation of neutrophils. Therefore, it performs a vital role in inflammation-related diseases, including pulmonary disease (Qin et al., 2017; Lee et al., 2019).

Histopathological examination of lung tissues showed serious degenerative changes with loss of lung architecture in the BLM-induced group evidenced by obvious congestion and dilatation along blood vessels, thickening in alveolar epithelium and smooth muscle, the massive number of inflammatory cells infiltration, interstitial edema, hyperplasia of bronchiole lining epithelium, as well as the noticeable increase in fibrous amount, in line with the results of previous works (Zhao et al., 2008; Guo et al., 2013; Yu et al., 2016; Zaghloul et al., 2019).

In contrast, these alterations were attenuated upon rutin administration, in harmony with previously reported results (Bai et al., 2020; Gao et al., 2013; Chen et al., 2014), showing the evident capacity of rutin to block the initial inflammatory response and emphasizing its potentiality against BLM-induced ALI.

Whence, thorough bioinformatics analyses were employed to investigate rutin’s molecular action mechanism. Among rutin target genes and ALI-related genes, 147 shared targets were identified. The GO and KEGG enrichment analyses revealed that the shared genes were distinctly enriched in some of the closely related BP, MF, and pathways to ALI, such as the response to hypoxia, inflammatory response, positive regulation of MAP kinase activity, C-C chemokine receptor activity, C-C chemokine binding, and HIF-1 signaling pathway.

Later, the PPI network characterized protein-protein interactions followed by screening the hub genes using different algorithms in the Cytoscape software, revealing *NFKB1* as the most remarkable. Two strategies were accordingly embraced; the first was predicting and determining the expression level of the upstream epigenetic miRNA regulators of *NFKB1*/*Nfkb1*, and the other was determining the *Nfkb1* gene and protein expression level alongside the gene and the protein expression levels of some of its downstream targets and related markers.

As important inflammatory regulators, significant alterations in miRNAs expression levels were found in ALI, suggesting their pivotal functional roles (Ferruelo et al., 2018). Herein, three databases (TargetScanHuman Release 8, miRTarBase, and DIANA-microT 2023) were interrogated to predict the upstream miRNAs targeting *NFKB1* in humans and rats, that revealed hsa-miR-9-5p and rno-miR-9a-5p as shared miRNAs. According to the PSRR web server, hsa-miR-9-5p appeared to be up-regulated by rutin, while the miRBase database showed 100% sequence congruity between the two miRNAs. Moreover, RNAhybrid was used to analyze the interaction of hsa-miR-9-5p and rno-miR-9a-5p with *NFKB1* and *Nfkb1* mRNAs, respectively, that showed a strong binding between the miRNAs and their target with a continuous base pairing at the seed region.

MiR-9 expression was downregulated in lung tissues of animals with ventilator-induced lung injury (Vaporidi et al., 2012; He et al., 2022). The same results were obtained by Xiao et al. (Xiao et al., 2023) in rats with LPS-induced ARDS. The authors found that miR-9 attenuated lung injury and alveolar hyper-coagulation. Further, miR-9 was negatively correlated with lung severity in ARDS patients, and the functional enrichment analysis revealed that its molecular mechanisms are closely related to fibrosis and inflammation (García-Hidalgo et al., 2022). This goes conjointly with the results of the current study that revealed a down-expression of rno-miR-9a-5p in lung tissues of the BLM-induced animals compared to the negative controls. Concurrently, treatment with rutin, at both doses, reversed the effect of BLM, resulting in an increased rno-miR-9a-5p expression level.

Because of its essential role in controlling each aspect of the inflammatory response, transcription factor NF-κB is considered a master regulator of inflammation (Zhang et al., 2017). Therefore, its aberrant regulation leading to detrimental inflammation is a major driver of many inflammatory diseases, including ALI (Ye et al., 2008). NF-κB is mainly expressed as a homo/heterodimeric complex comprising of RelA (p65), RelB, Rel (c-Rel), NF-κB1 (p50/p105), and NF-κB2 (p52/p100). The best characterized NF-κB dimer is p50-p65, which is activated by several exogenous and endogenous stimuli, translocating to the nucleus upon activation to induce the transcription of a wide array of genes (Alvira, 2014). *NFKB1*/*Nfkb1* gene encodes a single mRNA, from which p105 and p50 are co-translated. Further, the active p50 subunit is produced by procession of its p105 precursor molecule (Pereira and Oakley, 2008).

It is worth mentioning that in different animal models of induced ALI, the expression of the *Nfkb1* gene and the NF-κB p50 protein were upregulated in the injured lung tissues compared to their corresponding controls (Fei et al., 2019; Dong et al., 2020; Mitchell et al., 2020; Zhao et al., 2022). This is in congruence with the results of the present work demonstrating a significant over-expression of pulmonary *Nfkb1* gene and increased NF-κB p50 protein expression in the BLM-intoxicated group compared to the control group. Conversely, rutin intervention decreased the pulmonary *Nfkb1* gene and NF-κB p50 protein expression significantly, which would affect the assembly and synthesis of NF-κB complex. This is in line with previous reports that demonstrated the ability of rutin to downregulate the expression of *Nfkb1* and the p50 subunit both *in vivo* and *in vitro* (Su et al., 2019; Rana et al., 2022; Wang et al., 2023).

It is well known that an adequate balance of pro-inflammatory and anti-inflammatory factors is essential for limiting the detrimental effects of inflammation. COX-2 encoded by the *Ptgs2* gene is a crucial enzyme in controlling the production of inflammatory prostaglandins, and ample evidence suggests that NF-κB increases COX-2 expression due to the presence of two NF-κB binding sites in the *Ptgs2* promoter region (Tanabe and Tohnai, 2002). Further, NF-κB is believed to be a transcriptional activator of the pro-inflammatory IL-18. The latter enhances the secretion of IFN-γ from natural killer cells and activated T lymphocytes and thus is termed IFN-γ-inducing factor (Zhang et al., 2018). Additionally, it was indicated previously that NF-κB could directly stimulate the expression of IFN-γ, whose presence is a typical sign of inflammatory responses (Kauppinen et al., 2013). It is worth noting that the early inflammatory response, typically characterized by the recruitment of inflammatory cells to the lung and the release of chemokines and inflammatory factors, is one of the key determinants of BLM-induced ALI (Li et al., 2023; Wu and Wang, 2019; Abdelhady et al., 2023). At the same time, rutin possesses robust anti-inflammatory activity against many diseases, including lung injury (Tosun et al., 2021; Huang et al., 2016; Chen et al., 2023; Muvhulawa et al., 2022). This conforms with the results of the current work that revealed a marked elevation in the gene expression level of *Nfkb1*, *Ptgs2*, *Il18*, and *Ifng* and their protein products (NF-κB p50, COX-2, IL-18, and IFN-γ, respectively) in the BLM-induced group. Meanwhile, the treatment with rutin reverberated these changes.

IL-10 is a potent anti-inflammatory cytokine that confers immunosuppressive effects, mainly by down-regulating macrophage functions and reducing monocyte/macrophage production of pro-inflammatory cytokines (De Kozak et al., 2002). However, previous studies reported contradictory results regarding IL-10 levels in BLM-intoxicated rodents (Zaghloul et al., 2019; Li et al., 2023). In the current study, IL-10 gene expression and protein levels were significantly elevated in the BLM-induced group compared to the negative control group, which can be considered a host defense mechanism to suppress the release of the pro-inflammatory cytokines (Doughty et al., 1998). However, this endeavor failed to commensurate with the uncontrollable inflammatory process. On the other hand, the anti-inflammatory property of rutin was able to augment the gene expression and protein levels of IL-10.

Considering the preceding, a possible mechanism of rutin against BLM-induced ALI can be proposed. Via regulating the expression of rno-miR-9a-5p and its downstream target, *Nfkb1*, rutin suppresses the recruitment of neutrophils into the alveolar spaces, inhibits the induction of early-response cytokines and chemokines, and consequently, the alterations in the following inflammatory cascade events, relieving eventually BLM-induced ALI.

In conclusion, this work enlightens the potential effect of rutin against BLM-induced ALI based on network pharmacology and experimental validation. Rutin ameliorated BLM-induced ALI through a functional mechanism involving upregulation of miR-9a-5p impacting NF-κB mediated inflammation.

While the present findings and mechanistic rationale suggest a potential therapeutic utility of rutin against BLM-induced ALI, this study remains preliminary and has yet to be validated. Therefore, additional studies would lay a solid foundation for the therapeutic use of rutin. Further, more pharmacokinetic investigations are imperative to ensure sufficient absorption and distribution within the body. Finally, well-designed clinical trials are required to elaborate on the safety and therapeutic efficacy of rutin.

## Data Availability

The original contributions presented in the study are included in the article/Supplementary Material, further inquiries can be directed to the corresponding author.
